# Design and Implementation of a Portal for the Medical Equipment Market: MEDICOM

**DOI:** 10.2196/jmir.3.4.e32

**Published:** 2001-12-26

**Authors:** Stergios Palamas, Dimitris Kalivas, Ourania Panou-Diamandi, Cees Zeelenberg, Chris van Nimwegen

**Affiliations:** ^1^National Technical University of AthensBiomedical Engineering Laboratory9 Iroon Polytechniou Str., Computer Building15773 AthensGreece; ^2^Technological Education Institute of PiraeusDepartment of Electronics250 Thivon Str.12244 AthensGreece; ^3^Nederlandse Organisatie voor Toegepast Natuurwetenschappelijk Onderzoek (TNO)-Prevention and HealthDepartment of Technology in HealthcareZernikedreef 9, P.O. Box 22152301 ce LeidenNetherlands

**Keywords:** Electronic commerce, medical devices, equipment and supplies, Internet, CORBA, XML, RDBMS

## Abstract

**Background:**

The MEDICOM (Medical Products Electronic Commerce) Portal provides the electronic means for medical-equipment manufacturers to communicate online with their customers while supporting the Purchasing Process and Post Market Surveillance. The Portal offers a powerful Internet-based search tool for finding medical products and manufacturers. Its main advantage is the fast, reliable and up-to-date retrieval of information while eliminating all unrelated content that a general-purpose search engine would retrieve. The Universal Medical Device Nomenclature System (UMDNS) registers all products. The Portal accepts end-user requests and generates a list of results containing text descriptions of devices, UMDNS attribute values, and links to manufacturer Web pages and online catalogues for access to more-detailed information. Device short descriptions are provided by the corresponding manufacturer. The Portal offers technical support for integration of the manufacturers' Web sites with itself. The network of the Portal and the connected manufacturers' sites is called the MEDICOM system.

**Objective:**

To establish an environment hosting all the interactions of consumers (health care organizations and professionals) and providers (manufacturers, distributors, and resellers of medical devices).

**Methods:**

The Portal provides the end-user interface, implements system management, and supports database compatibility. The Portal hosts information about the whole MEDICOM system (Common Database) and summarized descriptions of medical devices (Short Description Database); the manufacturers' servers present extended descriptions. The Portal provides end-user profiling and registration, an efficient product-searching mechanism, bulletin boards, links to on-line libraries and standards, on-line information for the MEDICOM system, and special messages or advertisements from manufacturers. Platform independence and interoperability characterize the system design. Relational Database Management Systems are used for the system's databases. The end-user interface is implemented using HTML, Javascript, Java applets, and XML documents. Communication between the Portal and the manufacturers' servers is implemented using a CORBA interface. Remote administration of the Portal is enabled by dynamically-generated HTML interfaces based on XML documents.

A representative group of users evaluated the system. The aim of the evaluation was validation of the usability of all of MEDICOM's functionality. The evaluation procedure was based on ISO/IEC 9126 Information technology - Software product evaluation - Quality characteristics and guidelines for their use.

**Results:**

The overall user evaluation of the MEDICOM system was very positive. The MEDICOM system was characterized as an innovative concept that brings significant added value to medical-equipment commerce.

**Conclusions:**

The eventual benefits of the MEDICOM system are (a) establishment of a worldwide-accessible marketplace between manufacturers and health care professionals that provides up-to-date and high-quality product information in an easy and friendly way and (b) enhancement of the efficiency of marketing procedures and after-sales support.

## Introduction

The main objective for a European electronic marketplace for medical devices is to establish an environment hosting all the interactions of consumers (health care organizations and professionals) and providers (manufacturers, distributors, and resellers of medical devices). This objective is to be accomplished by concentrating the forces of organizations that are interested in globalization of the medical-products market. This has become necessary because the growth of the medical market requires introducing modern technologies for developing products and for establishing marketing policies. Our objective was to build a service that relies on seamless information exchange on the Internet to facilitate business relations (marketing, sales, and after-sales processes).

The MEDICOM (Medical Products Electronic Commerce) system is an Internet-based system that includes a unique Portal, which more or less acts as the yellow pages (a list of business and professional firms alphabetically by category; typically part of a telephone directory) for finding both products and providers. Its main advantage is fast, reliable, up-to-date information retrieval that eliminates all unrelated content that a general-purpose search engine would retrieve. The Portal accepts the end-user requests and generates a list of results containing text descriptions of devices, UMDNS (Universal Medical Device Nomenclature System) Attribute Values, and links to the providers' servers (for Web pages, online catalogues, and post-market surveillance systems) for access to more-detailed information in a way that is transparent to end-user customers. The post-market surveillance system collects information about adverse incidents; information on these incidents needs to be exchanged among competent authorities, health care institutions, and manufacturers, to deal with the consequences of the incidents and prevent reappearance of the same incidents.

The requirements of the medical-devices electronic-commerce community along with the technical requirements of the MEDICOM system have been analyzed within the framework of the MEDICOM project [1]. The European Commission, under the ESPRIT program, supported the project.

The end users of the MEDICOM system are clinicians; doctors; hospital administrative staff; clinical engineers; and in general everyone who uses medical devices, is involved in purchasing medical devices, or technically supports medical devices. The end-user requirements are, in summary:

- Advanced search and retrieval of structured and up-to-date information on medical devices of multiple manufacturers
- Friendly-and-informative multimedia presentations of medical products
- Presentation of complex equipment using virtual reality techniques
- Efficient after-sales support (for example, reporting incidents and technical assistance).

The medical equipment providers (manufacturers, distributors, and resellers) form the second user group of the system. Their main requirements are:

- Internet presence with Hypermedia Product Catalogues
- A secure communication channel with their customers for Post Market Surveillance (PMS)
- Access to user profiling and user-related statistical information
- Modularized service architecture which enables distribution of manufacturers' servers
- Capability of hosting a manufacturer's server at an Internet Service Provider's site
- Cost effectiveness.

Technical requirements are:

- Interoperability and easy integration of the MEDICOM platform with existing manufacturer infrastructure
- Consideration of all security issues about data integrity, confidentiality, and authentication
- Conformity of the technical implementation to existing standards.

In addition to the user requirements and technical requirements, some MEDICOM system-design issues have been considered:

- Even though on-line sales transactions and payment systems are not a usual practice in the current medical-device procurement process, it is anticipated that this will change. Therefore, incorporation of sales transactions and payment systems should be foreseen.
- An agreed and widely-used nomenclature system for the identification and description of medical devices in general terms is needed, to allow collation and data exchange across Europe.

Since UMDNS was already widely used in Europe and the European Commission had adopted UMDNS as an interim standard, it was decided to use UMDNS as the classification basis throughout the MEDICOM system [2].

## Methods

### System design.

**Figure 1:**
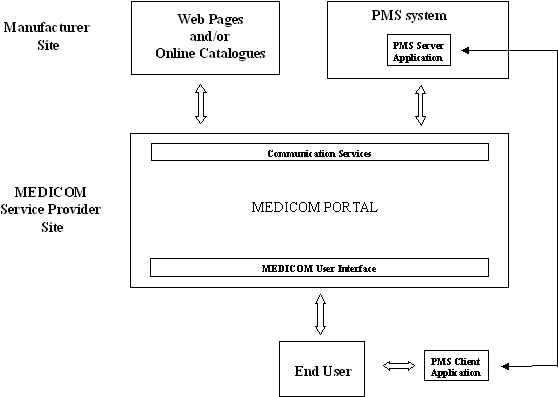
The MEDICOM system architecture

The MEDICOM system has been implemented using a modularized and distributed architecture. The main components of the system are shown in Figure 1. The Portal provides the end-user interface, handles system management, and ensures database compatibility throughout the system. It also provides necessary communication services, technical support for MEDICOM integrators, and additional services for the end users.

The MEDICOM Portal provides access to the whole system and coordinates the operation of the servers (catalogue and PMS servers) hosted by the manufacturers. The Portal hosts a database composed of 2 parts: (a) the Common Database, which stores a set of encoded parameters that will be included on all servers and (b) the Short Description Database, which contains the short descriptions of all medical products registered to the system.

The Portal is responsible for maintaining, altering on demand, distributing across the subsystems, and ensuring the consistency of the information in the Common Database. The structure of the Common Database includes the following data (see the next 2 paragraphs for explanations of some of the terms): Supported Languages, Geographic Regions, UMDNS Codes, UMDNS Attributes, Predefined UMDNS Attribute Values, Manufacturers, Authorized Representatives and Distributors, Incident Reports Classification Codes, Healthcare Institutions with PMS Client Systems, Quality Certificates of Medical Devices, Organizations Classification Codes, Specialism Codes of End Users, Producers Mailing Lists, End-users Job Titles, and Authorities.

The UMDNS Code is a unique identifier for each product category. Each product category (that is, each UMDNS code) is characterized by a set of features (for example, the operational voltage and the resolution). In the UMDNS, these features are called UMDNS Attributes. The set of values of each attribute are the attribute values. An example is the Device Category *Scanners, Ultrasonic, General Purpose*, which is assigned the unique UMDNS code 15976. For this device category there are 5 attributes (features) defined in the UMDNS coding system: System, Doppler/C.V.I., Transducers (arrays), Application, and Accessories. These attributes are intended to be the most important features of this device category. Each of the attributes has one or more values. The values can be restricted to a predefined set of values or can be unrestricted. For example, the attribute Transducers has a set of predefined values (Phased, Linear, and Curved Linear); the attribute Application can take any value. Using the attribute and attribute values, the UMDNS codification ensures that for each device category a common set of important feature characteristics will be supplied.

Authorities (Competent Authorities and Notified Bodies) receive reports on serious incidents involving medical devices. The reports are submitted by the manufacturers or the health care institutions. Generally, the authorities have to monitor the manufacturer's investigation and intervene when necessary.

The Short Description Database contains summarized descriptions of medical devices, whose extended descriptions are presented by the manufacturer's servers. The Short Description includes a text description, the manufacturer and/or authorized representatives and/or distributors, the UMDNS code, important product features, and links to the corresponding HTML pages of the manufacturers' servers (where extended information exists). The contents of the Short Description Database will be updated as a result of a manufacturer's-server request.

To satisfy the end-user requirements the Portal provides the end-user with the following services:

- **Profiling and registration:** The Portal maintains user profiles and handles user-authentication and security issues. In addition, it provides user-related information to the manufacturers on request.
- **Product Searching mechanism:** Perhaps the most useful service. Searching can use one or more of the following criteria:

-
  - Manufacturer: useful when searching for the whole range of products of one manufacturer.
  - Distributor: useful when searching for products distributed by a specific distributor.
  - Product Category (based on the UMDNS classification): used when searching for a specific product category.
  - Product Name: used when searching for a specific product.
  While searching, the user can define some parameters that narrow the resulting set of devices, such as the language of the provided information or the region where the requested products are available. The Portal, based on the searching parameters, performs a query in the local Short Description Database, and generates a list of matching products along with all the data that are stored in the Portal for each one of these products. The resulting set contains links to remote manufacturers' servers where the user can access more information about the specific product. Depending on the specific manufacturer, this information may include multimedia presentations and virtual exhibitions of complex equipment.
- **Free-Text Searching:** A search engine will allow the users to perform free-text queries on a selected set of Web sites (eg, the Web sites of the participating manufacturers). Restricting the user's query to a small number of sites that are highly related to the MEDICOM end-user's interests increases the relevance of the links that result from the user's query. This service is implemented with the Netscape Compass server (Version 3). Compass can index HTML, ASCII, Microsoft Word, Microsoft PowerPoint, Adobe PDF (Portable Document Format), and various other document formats on local or remote WWW (World Wide Web) and FTP (File Transfer Protocol) sites.
- **Bulletin Board:** The Portal hosts a bulletin board covering a wide range of topics relative to the end-users' interests. The end user will have the option to search through the posted articles, contact the authors, and initiate a new discussion thread.
- **Literature and Standards:** Literature and standards about specific products or product categories is stored in the Portal and end users can search though it.
- **On-line information for the MEDICOM system and Special Messages or Advertisements from Manufacturers.**

In addition to providing services to the end users, the Portal provides the following services to the manufacturers:

- **Technical Specifications for the MEDICOM Integrators:** The Portal will provide specifications and technical support, if necessary, for integration of proprietary subsystems with the MEDICOM system. There is a strong possibility that a manufacturer has already-developed Web pages and online catalogues, which the MEDICOM system should be able to incorporate.
- **Browsing and Updating of the Common Database:** The updates on this information affect the whole system, thus the Portal administrator moderates them.
- **Browsing and Updating of the Short Description Database:** Each manufacturer has access only to the data related to its products.
- **Access to system statistics and user profiling.**

Modularity, platform independence, and interoperability characterize the design of the Portal. Internet-standard technologies have been utilized wherever possible. The first version was developed for Windows NT and it is currently ported to HP-UNIX (Hewlett-Packard UNIX). All the data across the MEDICOM system are structured and maintained in databases. The Oracle 8 RDBMS has been used to develop the Portal databases. The end-user interface is a combination of static and dynamic HTML pages. Javascript code, which is executed by the user's browser, parses the XML (eXtensible Markup Language) data and displays the data on the browser [3]. Separation of formatting information and data through the use of XML has a strong benefit. The code, which formats the data, is transferred only once to the user's browser. From that point on, the browser receives only XML documents, which are parsed and presented. Communication and data exchange between (a) the Portal and (b) the remote Web sites and on-line catalogues of the medical equipment manufacturers and suppliers are based on CORBA (Common Object Request Broker Architecture) [4]. Java has been used for application development, enhancing the portability of code to different platforms [5,6].

### System evaluation.

The objective of the evaluation was to validate the usability of the complete MEDICOM functionality. To evaluate the system effectiveness and efficiency, representative users were asked to complete typical tasks. The evaluation procedure was based on ISO/IEC (International Organization for Standardization/International Electrotechnical Commission) 9126, Information technology - Software product evaluation - Quality characteristics and guidelines for their use [7].. Additionally, simple questionnaires were used to assess user satisfaction.

The users of the system were divided into 3 groups:

- End Users (hospital managers, clinicians, clinical engineers, and procurement office administrators)
- Manufacturers (IT [Information Technology] specialists)
- Service Provider (operator of the Portal)
  We intend to provide worldwide service through one organization - TNO (Toegepast Natuurwetenschappelijk Onderzoek [Netherlands Organization for Applied Scientific Research]), so there Service Provider, rather than Service Providers, is listed. However, there is the possibility of, for example, different portals in each country with each portal operated by a different service provider.

End-user functionality was evaluated by AUSL (Azienda Unita' Santaria Locale Di Modena) of Modena, which is the health care organization responsible for public service in the province of Modena, Italy. Manufacturer functionality was evaluated by Esaote S.p.A. (a medical-device company). Service-provider functionality was evaluated by the Prevention and Health Division of TNO.

## Results

The overall user evaluation of the MEDICOM system was very positive. The MEDICOM system was characterized as an innovative concept that brings significant added value to the process of medical-equipment commerce.

Searching for a product in a specific product category resulted in a list of matching products that was highly representative of the actual market. The amount of information in the short description of each product was considered well chosen. The searching procedure was easy to use, even by persons without specific computer experience. The on-line help was often used and the information returned was adequate. The resulting list of matching products never contained products that should not be included (that is, that did not match the searching criteria). In comparison to the searching procedures that were used up to then the MEDICOM system proved to be more effective, more efficient and more user friendly.

## Discussion

Researching the overall competition to MEDICOM showed that no company offers the complete set of MEDICOM services. Some companies only present basic company information or list their products, with neither search nor ordering options. Some companies present only part of the possible product mix, or they supply products from only one manufacturer or from a few manufacturers. Most companies offer only basic customer service or give their telephone number and/or e-mail address. Although there are Web sites where it is possible to order products made by many manufacturers, these Web sites are not strong potential competitors to MEDICOM, since they are not portals and their product mix is not as wide as MEDICOM's product mix.

The MEDICOM Portal targets the manufacturers of medical devices by stressing the importance of the Internet as a mean of providing information and promoting their products. The Internet is a new and cost-effective way of diffusing product information, offering technical and after sales support, providing information about new products and services, evaluating the competition, finding new distributors and suppliers, and reaching global customers.

For health-care professionals, MEDICOM provides a wide range of accurate information about new medical products and services. It will increase health-care professionals' knowledge about the market and it will improve communication between them and the manufacturers for after-sales and technical support. Most importantly, it will facilitate the involvement of all actors in the purchasing process for new medical equipment by providing a wide range of accurate information, by giving access to detailed technical features and commercial conditions on-line, and by offering an overview of the products' competition.

## Abbreviations

AUSL: Azienda Unita' Santaria Locale Di Modena
CORBA: Common Object Request Broker Architecture
ISO/IEC: International Organization for Standardization/International Electrotechnical Commission
IT: Information Technology
MEDICOM: Medical Products Electronic Commerce
PMS: Post Market Surveillance
RDBMS: Relational Database Management Systems
TNO: Toegepast Natuurwetenschappelijk Onderzoek (Netherlands Organization for Applied Scientific Research)
UMDNS: Universal Medical Devices Nomenclature System
XML: eXtensible Markup Language
